# Transient ciliochoroidal detachment after microhook ab interno trabeculotomy: Its frequency and potential risk factors

**DOI:** 10.3389/fmed.2022.1028645

**Published:** 2022-11-04

**Authors:** Fumiya Miyako, Kazuyuki Hirooka, Hiromitsu Onoe, Naoki Okada, Hideaki Okumichi, Yoshiaki Kiuchi

**Affiliations:** Department of Ophthalmology and Visual Science, Hiroshima University, Hiroshima, Japan

**Keywords:** microhook, trabeculotomy, ciliochoroidal detachment, central corneal thickness, intraocular pressure

## Abstract

**Purpose:**

To investigate ciliochoroidal detachment (CCD) frequency and risk factors after performing microhook ab interno trabeculotomy (μLOT).

**Methods:**

A retrospective evaluation of 62 eyes of 62 patients who underwent μLOT and were subsequently examined by anterior-segment optical coherence tomography (AS-OCT) found CCD at 1 day, and 1 and 2 months after surgery.

**Results:**

In the 62 patients (mean age 67.3 ± 13.9 years), AS-OCT detected CCD in 18 eyes (29%) at 1 day after surgery, which disappeared within 1 month. Comparisons between the CCD vs. the non-CCD group showed the mean IOPs were 11.7 ± 1.5 mmHg vs. 14.4 ± 1.0 mmHg at day 1 (*P* = 0.13), 12.2 ± 1.1 mmHg vs. 14.8 ± 0.7 mmHg at day 7 (*P* = 0.06), 12.2 ± 0.7 mmHg vs. 12.9 ± 0.5 mmHg at 1 month (*P* = 0.48), and 11.3 ± 0.7 mmHg vs. 12.7 ± 0.5 mmHg at 2 months (*P* = 0.09). For postoperative IOP, there were no significant differences observed. After undergoing μLOT, multiple regression analysis demonstrated that the CCD development might be influenced by the presence of a thinner central corneal thickness.

**Conclusion:**

Approximately one-third of all patients exhibited CCD after μLOT. A thinner central corneal thickness was found to be a risk factor for developing CCD.

## Introduction

Trabeculotomy is used to reduce the aqueous humor outflow tract resistance. When using this method, the reticular structure of the trabecular meshwork and the inner wall of Schlemm’s canal are cleaved (1, 2). However, intraocular pressure (IOP) levels are only decreased by trabeculotomy ab externo to the middle teens, with single digit IOPs rarely achieved (3). A safer and less traumatic surgical intervention, minimally invasive glaucoma surgery (MIGS), has been developed for patients with mild to moderate glaucoma or patients who have been found to be intolerant to standard medical therapy (4). The three types of MIGS devices are the trabecular, suprachoroidal and subconjunctival based (5). There have been several attempts to design a new way of reducing the trabecular meshwork resistance from within the anterior chamber, which has led to the utilization of the trabectome, Kahook Dual Blade (New World Medical, Rancho, Cucamonga CA), and microhook (Inami & Co., Ltd., Tokyo, Japan), in conjunction with 5–0 nylon sutures (1, 6–8). Trabecular outflow through Schlemm’s canal is improved by these trabecular based devices, with these changes leading to the aqueous humor from the trabecular outflow tract flowing into the episcleral venous system. As a result, drops in the IOP below the scleral venous pressure, which ranges from 7.6 to 11.4 mmHg, should not occur when using this trabeculotomy method (9). However, after ab interno trabeculotomy, there have been reports of cases exhibiting transient low IOPs (less than 5 mmHg) (10). It has been speculated that ciliochoroidal detachment (CCD) could be responsible for low IOPs that are observed immediately after ab interno trabeculotomy (11) or after 360-degree suture trabeculotomy ab interno (S-LOT) (12), with CCD observed in 42∼48% of these cases. The differences observed when cleaving open the trabecular meshwork and then excising it may be associated with the approach and specific instrument used, with these potentially responsible for causing the alterations noted in the CCD rate.

In this investigation, we examined microhook ab interno trabeculotomy (μLOT) using anterior-segment optical coherence tomography (AS-OCT) and tried to determine if CCD immediately occurred after the procedure in addition to evaluating risk factors for CCD development.

## Materials and methods

### Patients

Between January and December of 2021, this retrospective study evaluated glaucoma patients who underwent μLOT at Hiroshima University Hospital. The study protocol was approved by the Institutional Review Board of the Hiroshima University. In accordance with the principles outlined in the Declaration of Helsinki, all subjects provided written informed consent in addition to providing standard consent for surgery.

Patients were excluded if they had previously undergone intraocular surgery, with the exception for those who had undergone cataract surgery. In order to be included in the study, all patients had to be ≥ 20 years of age in addition to having no history of other significant ocular diseases. For patients who underwent treatment in both eyes, the analysis used only the first eye treated. This study enrolled 81 eyes, with 19 eyes subsequently excluded from the analysis due to the fact they did not meet the required 2-month postoperative follow-up period (2 eyes) or the AS-OCT was not performed because of oblivion (17 eyes). In total, 62 of 81 eyes (77%) were analyzed in this study (Table 1).

**TABLE 1:** Clinical characteristics of study patients.

Age (years), mean (*SD*)	67.3 (13.9)
Gender (M/F)	23/39
Type of glaucoma	
POAG	23
PACG	29
Exfoliation glaucoma	6
JOAG	4
Central corneal thickness (μm), mean (*SD*)	503 (36)
Axial length (mm), mean (*SD*)	24.7 (2.2)
Mean Deviation (dB), mean (*SD*)	−8.96 (6.46)
Surgical procedure	
Combined	48
Single	14
Preoperative IOP (mmHg), mean (*SD*)	16.0 (6.1)
No. IOP-lowering medications, mean (*SD*)	2.5 (1.3)

M, male; F, female; POAG, primary open-angle glaucoma; PACG, primary angle closure glaucoma; JOAG, juvenile open-angle glaucoma; IOP, intraocular pressure.

### Surgical procedure

Details of the surgical technique have been described previously (13, 14). During the μLOT procedure, the microhook was intraocularly inserted through a temporal corneal incision. After visualizing the nasal trabecular meshwork using a Hill surgical gonioprism (Ocular Instruments Inc., Bellevue, WA), the microhook (Inami & Co., Ltd., Tokyo, Japan) was used to make a 120° incision in the nasal trabecular meshwork and the inner wall of Schlemm’s canal. If cataract surgery was being performed at the same time in the patients, a 2.75 mm temporal corneal incision was made for the cataract extraction and intraocular lens implantation prior to starting the μLOT procedure.

Starting on the first postoperative day, patients were instructed to use antibiotic [1.5% levofloxacin (Nipro Corporation, Osaka, Japan)], corticosteroid [0.1% fluorometholone (Santen Pharmaceutical, Osaka, Japan)] and 2% pilocarpine (Santen Pharmaceutical, Osaka, Japan) eye drops for 3–4 weeks three times daily. Non-steroidal anti-inflammatory [nepafenac (Novartis Pharma, Basel, Switzerland)] eye drops were also administered for 3–4 weeks three times daily in patients who underwent combined surgery. IOP-lowering medications were restarted in accordance with the postoperative IOP.

### Clinical examination

Gonioscopy, slitlamp examination, IOP measurement with Goldmann applanation tonometer, uncorrected and best-corrected visual acuity with Landolt ring chart at 5 m were performed preoperatively in all patients. In addition, all of these patients also underwent axial length measurement by partial laser interferometry (IOL-master; Carl Zeiss Meditec, Dublin, CA), central corneal thickness (CCT) measurement with specular microscope (Topcon SP-3000; Topcon Corporation, Tokyo, Japan), and standard automated perimetry (Humphrey Visual Field Analyzer using the 24–2 Swedish Interactive Thresholding Algorithm Standard testing protocol; Carl Zeiss Meditec, Dublin, CA).

Follow-up examinations were scheduled at 1, 3, and 7 days, and 1 and 2 months. IOP measurements and slitlamp examinations were performed at each follow-up. Visual acuity was assessed at 7 days, 1, and 2 months after surgery. AS-OCT (CASIA2; Tomey, Nagoya, Japan) examinations were performed in all patients at postoperative day 1, and months 1 and 2.

### Anterior-segment optical coherence tomography

**FIGURE 1:**
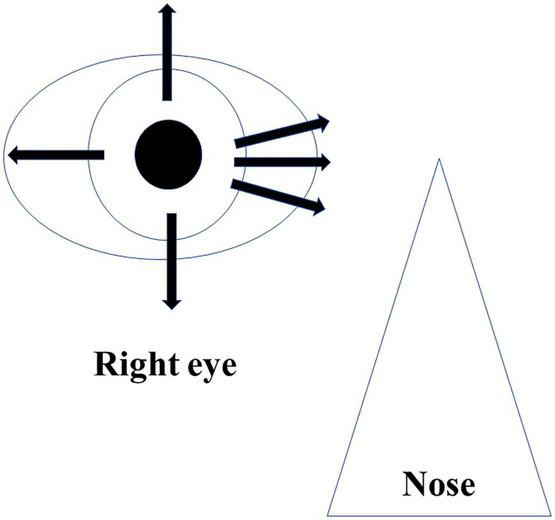
Imaging protocol of AS-OCT. Imaging was performed independently in each of the four quadrants (superior, inferior, nasal, and temporal), as well as 30° superior and inferior at the nasal area.

**FIGURE 2:**
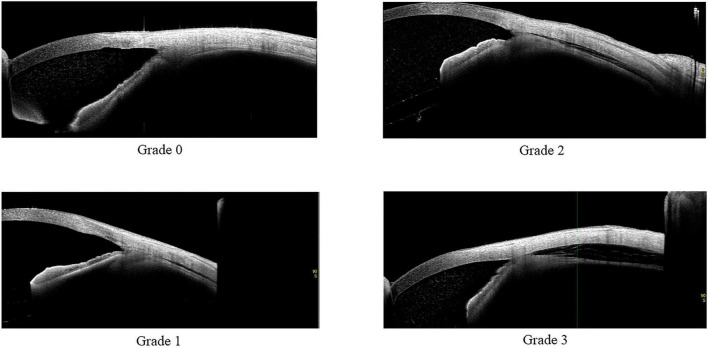
Classifications of ciliochoroidal detachment. Ciliochoroidal detachment was postoperatively observed.

AS-OCT was utilized for the CCD investigations after the surgery. During the imaging, patients were placed in a seated position and then asked to look toward the opposite side. During the evaluation, three nasal directions, the superior and inferior, and the temporal sides were examined (Figure 1). Classification of the CCD severity was defined based on the maximum CCD observed among the AS-OCT images (15). The defined classifications included grade 0 (no sign of CCD), grade 1 (slit-like, with CCD less than half of the ciliary body thickness), grade 2 (band-like, with CCD at least half of the ciliary body thickness), and grade 3 (obvious, with CCD greater than the ciliary body thickness) (Figure 2). The CCD and non-CCD groups at postoperative day 1 were defined as eyes with CCD (grade 1–3) and eyes without CCD (grade 0), respectively. Three ophthalmologists (F.M., K.H., and H.O.) who were masked to the clinical data assessed the AS-OCT images and determined the CCD presence.

### Statistical analysis

Statistical analyses were conducted using JMP software version 16 (SAS Inc., Cary, NC). Comparisons of the age, gender, axial length, CCT, surgical procedure (combined or single), visual field mean deviation, glaucoma type, preoperative IOPs, and preoperative IOP-lowering medications between the groups were performed using an unpaired *t*-test or χ^2^-test. Postoperative IOPs were analyzed using the multivariate analysis of variance (MANOVA). Factors associated with the CCD were determined by a multiple regression analysis. Factors tested included: age, gender, axial length, CCT, surgical procedure (combined or single), preoperative IOP, and glaucoma type (exfoliation glaucoma or others). As shown by the univariate analysis, multivariate factors were selected from the variants having a probability value of less than 0.2. All data are presented as the mean ± standard deviation (SD). *P*-values less than 0.05 were considered to indicate statistical significance.

## Results

In total, 18 eyes (29.0%) (CCD group) had grade 1–3 CCD and 44 eyes (non-CCD group) had no CCD at postoperative day 1 (Table 2). For the maximum CCD grade, 11 eyes (61.1%) were grade 1, 3 eyes (16.7%) were grade 2, and 4 eyes (22.2%) were grade 3. CCD was observed in all quadrants in 3 eyes (16.7%), 3 eyes had (16.7%) CCD in three quadrants (nasal, inferior, and superior), and CCD was found in one quadrant (nasal) in 12 eyes (66.7%).

**TABLE 2:** Clinical characteristics of study patients with or without CCD at postoperative day 1.

	CCD group (*n* = 18)	Non-CCD group (*n* = 44)	*P*-value
Age (years), mean (*SD*)	70.4 (3.3)	66.0 (2.1)	0.26
Gender (M/F)	9/9	14/30	0.18
Type of glaucoma			0.14
POAG	4	19	
PACG	11	18	
Exfoliation glaucoma	3	3	
JOAG	0	4	
Central corneal thickness (μm), mean (*SD*)	490 (40)	509 (33)	0.07
Axial length (mm), mean (*SD*)	24.2 (2.4)	24.8 (2.1)	0.37
Mean deviation (dB), mean (*SD*)	−8.71 (1.58)	−9.05 (0.99)	0.86
Surgical procedure			0.48
Combined	15	33	
Single	3	11	
Preoperative IOP (mmHg), mean (*SD*)	16.3 (1.4)	15.8 (0.9)	0.76
No. IOP-lowering medications, mean (*SD*)	2.6 (0.3)	2.5 (0.2)	0.68

CCD, ciliochoroidal detachment; M, male; F, female; POAG, primary open-angle glaucoma; PACG, primary angle closure glaucoma; JOAG, juvenile open-angle glaucoma; IOP, intraocular pressure.

**FIGURE 3:**
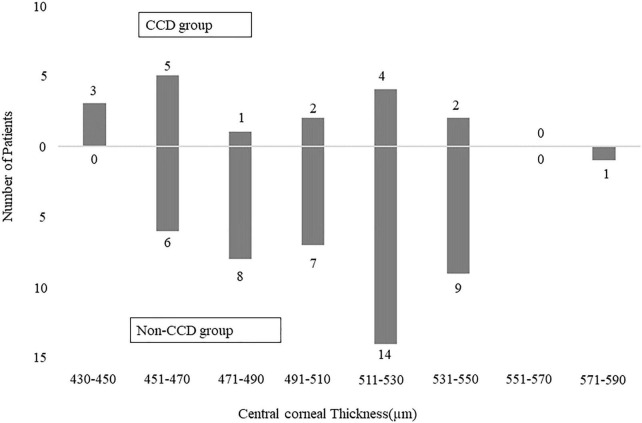
Bar graph showing the number of patients of CCD group and non-CCD group in each central corneal thickness.

Table 2 shows the comparisons of the age, gender, types of glaucoma, CCT, axial length, visual field mean deviation, surgical procedure, preoperative IOP, and number of IOP-lowering medications between the groups with and without CCD. A total of 50% eyes in patients with exfoliation glaucoma had CCD. Analysis showed that it was significantly more likely for a thinner CCT to cause CCD (*P* = 0.049) (Table 3). Although combined cataract surgery with canal-based glaucoma surgery may alter the outcome, our current study determined that combined cataract surgery did not exhibit any influence on the overall outcome. Figure 3 shows the number of eyes of CCD group and non-CCD group in each CCT.

**TABLE 3:** Multivariate analysis of factors associated with CCD.

	Multiple logistic regression		
	
Factors	Odds ratio	95% CI	*P*-value
Gender (Female)	2.306	1.035–0.187	0.18
Type of glaucoma (exfoliation glaucoma)	2.399	0.502–1.373	0.34
Central corneal thickness	0.024	−38.078 to −1.201	0.049

CCD, ciliochoroidal detachment; CI, confidence interval.

**FIGURE 4:**
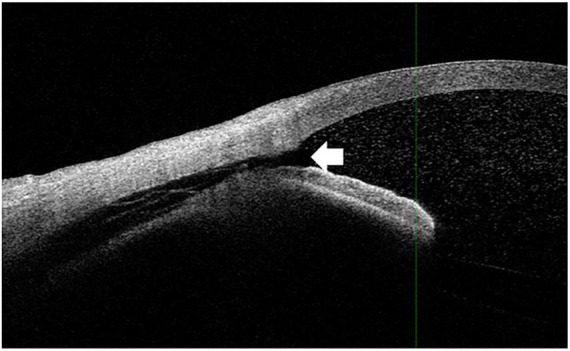
The connection between the anterior chamber and CCD. The white arrowhead indicates that the CCD was connected to the anterior chamber. The imaging shown in this figure was performed in the nasal quadrant.

Both the CCD and non-CCD groups exhibited similar postoperative IOP values through postoperative month 2 (MANOVA, *P* = 0.25) (Table 4). At month 2, the IOP-lowering medication scores were 0.67 ± 0.26 in the CCD group and 0.73 ± 0.17 in the non-CCD group (*P* = 0.86). A connection was observed for only the nasal quadrant between the anterior chamber and the CCD (connection group) in 11 of 18 eyes (61.1%) in the CCD group (Figure 4). Although both the connection and non-CCD groups exhibited similar postoperative IOP values through postoperative month 2 (MANOVA, *P* = 0.27), there was only a significantly lower IOP in the connection group at postoperative day 7 as compared to the non-CCD group (Table 5).

**TABLE 4:** Time course of IOP in eyes with or without CCD.

	CCD (*n* = 18)	Non-CCD (*n* = 44)	*P*-value
Preoperative	16.3 ± 1.4	15.8 ± 0.9	0.76
Day 1	11.7 ± 1.5	14.4 ± 1.0	0.13
Day 3	11.5 ± 1.4	13.6 ± 1.0	0.23
Day 7	12.2 ± 1.1	14.8 ± 0.7	0.06
Month 1	12.2 ± 0.7	12.9 ± 0.5	0.48
Month 2	11.3 ± 0.7	12.7 ± 0.5	0.09

IOP, intraocular pressure; CCD, ciliochoroidal detachment.

**TABLE 5:** Time course for the IOP in the connection group eyes (eyes with a connection between the anterior chamber and the CCD) and in the non-CCD group eyes.

	Connection (*n* = 11)	Non-CCD (*n* = 44)	*P*-value
Preoperative	16.1 ± 1.8	15.8 ± 0.9	0.90
Day 1	10.9 ± 1.9	14.4 ± 1.0	0.10
Day 3	10.0 ± 1.8	13.6 ± 1.0	0.09
Day 7	10.2 ± 1.3	14.8 ± 0.7	0.002
Month 1	12.3 ± 0.9	12.9 ± 0.5	0.57
Month 2	11.2 ± 0.9	12.7 ± 0.5	0.12

IOP, intraocular pressure; CCD, ciliochoroidal detachment.

With regard to postoperative complications, we observed hypotony (IOP ≤ 5 mmHg) in one eye in each group.

## Discussion

The present study investigated CCD presence in patients after undergoing the μLOT procedure. In 18 eyes (29.0%), postoperative CCD was detected at postoperative day 1, with the CCD disappearing within 1 month. Analysis demonstrated that a thinner CCT was associated with the CCD.

For postoperative IOP, there were no significant differences observed between the CCD and non-CCD groups in the current study. Akagi et al. (11) reported that the postoperative IOPs at all follow-up periods were lower in the CCD group than in the non-CCD group. On the other hand, Sato et al. (12) reported that the postoperative IOP value through postoperative month 12 were similar in both the CCD and non-CCD group, except the postoperative day 1.

After ab interno trabeculotomy, it remains unclear as to how CCD occurs. While comparisons of the connection and non-CCD groups showed there were no significant differences in the postoperative IOP between the CCD and non-CCD groups, on postoperative day 7 there was a significantly lower IOP in the connection group. It is our speculation that these findings suggest that enhancement of the uveoscleral outflow can lead to transient CCD. With regard to the postoperative CCD, it is possible that this could be due to postoperative hypotony. Previous studies have reported that CCD was associated with postoperative hypotony due to the overfiltration that was present after trabeculectomy (15), or wound leakage that occurred after cataract surgery (16). During the follow-up period in our current study, hypotony occurred in only one of the eyes and was less than 5 mmHg, and we found that there was no postoperative wound leakage in any of our cases. Based on our current findings, the cause of CCD is unlikely to be due to postoperative hypotony. Other studies have suggested that another possible cause of postoperative CCD could be due to ocular inflammation such as scleritis or Vogt-Koyanagi-Harada diseases (17, 18). However, in all of our cases, we did not find any severe inflammation in the conjunctiva, sclera, uvea, anterior chamber, or vitreous. Therefore, the main mechanism of CCD is unlikely to be due to surgical induced inflammation. Cyclodialysis can be caused by strong forces that occur along the longitudinal ciliary muscle, which leads to its separation from the scleral spar (19). Thus, one possible mechanism of CCD could be associated with the creation of an iatrogenic cyclodialysis cleft, which was a possible complication of our surgical technique. Alternatively, it is possible that a mechanism similar to the one that has been found after postoperative exudative choroidal detachment (ocular hypotension, inflammation) could be present, although this is less likely. Furthermore, intraoperative peeling of the trabecular meshwork by microhook could iatrogenically cause the CCD.

Our current study found that within 1 month, the CCD had disappeared in all of the cases. Similarly, Sato et al. (12) found that by month 3, all of the evaluated cases showed disappearance of the CCD. Using AS-OCT, Akagi et al. (11) at postoperative day 3 and 10 assessed the CCD. If CCD was found in any of the AS-OCT images at postoperative day 10, patients underwent a subsequent AS-OCT evaluation at 1 month. In our study, although at 1 month after the surgery we found that there were 4 eyes with CCD, it remains unknown as to when the CCD disappeared in these eyes. Another study demonstrated the presence of CCD in all 4 eyes (0.6%) that developed persistent hypotony (19). Moreover, it has also been reported there was an association between higher CCD grades and lower IOPs (11). In our current study, we found that at postoperative day 7 there was a significantly lower IOP in the connection group as compared to the non-CCD group. It is possible that the CCD related to the microhook surgery that was found in 11 eyes had a connection between the anterior chamber and CCD, with the other 7 eyes associated with a different mechanism. Sato et al. (12) found that 6 out of 21 eyes (28.6%) exhibited a connection between the anterior chamber and the CCD. In addition, in another study that examined the connection between the anterior chamber, they reported detecting CCD in 3 out of 4 eyes that developed persistent hypotony (20). Thus, overall these results suggest that after ab interno trabeculotomy, it is important to evaluate patients and determine whether CCD has occurred and if there is a connection between the anterior chamber and the CCD.

Results of our current study demonstrated that a thinner CCT was a risk factor for developing CCD. Akagi et al. (11) additionally reported finding a thinner CCT in the CCD group (505.9 ± 35.8 μm) as compared to that found in the non-CCD group (533.9 ± 39.1 μm). Furthermore, it has also been shown that the ciliary body can be distinct from the scleral spar due to severe blunt force, thereby causing CCD (19). However, if the CCT is thinner, it is our assumption that due to the weak connection between the ciliary body and scleral spar, even a mild to moderate blunt force could easily cause CCD. It has been reported that the CCT and corneal hysteresis are moderately correlated in patients with primary open-angle glaucoma and ocular hypertension (21). Thus, a low corneal hysteresis might reflect an increased biomechanical susceptibility or evolving tissue remodeling in these eyes (21). However, at the present time it still remains unclear as to the distinct relationship between the development of CCD and the CCT thickness. In order to clarify this relationship, further studies with larger sample sizes will need to be undertaken.

It has also been suggested that after gonioscopy-assisted transluminal trabeculotomy, it is possible that supraciliary fluid could be detected during anterior segment imaging without CCD and low IOPs (22). In these patients, surgery-induced inflammation might be an underlying factor (23). However, in our current study, the non-CCD patients did not exhibit any isolated supraciliary fluid.

## Limitations

The results of our current study need to be taken into consideration in conjunction with the potential limitations. First, with the exception for patients with primary angle closure glaucoma (PACG), we did not preoperatively perform AS-OCT. In the PACG patients, imaging was performed in each of the four quadrants (superior, inferior, nasal, and temporal) prior to the surgery in order to evaluate the anterior segment. In these patients, CCD was not observed before the surgery. While Sato et al. (12) evaluated patients prior to surgery and found no CCD, Akagi et al. (11) reported that 1 out of 37 eyes (2%) were found to have CCD prior to undergoing surgery. Thus, while the presence of CCD prior to surgery cannot be definitively ruled out, it is our belief that the chance of CCD being present before surgery is extremely rare. Our evaluation of CCD at day 1 and at months 1 and 2 is also another limitation. After performing LOT ab interno using trabectome in 33 eyes with open-angle glaucoma (OAG), Akagi et al. (11) reported that on postoperative day 3, they found that there were 14 eyes (42.4%) with CCD. Sato et al. (12) performed S-LOT in 44 eyes with OAG and reported that 21 eyes (47.7%) had CCD at postoperative day 7. CCD was initially detected in 14 of these 22 eyes on postoperative day 1, with CCD also seen on postoperative day 3 in 5 eyes, and on postoperative day 7 in 2 eyes (12). In our current study, we found CCD on postoperative day 1 in 18 out of 62 eyes (29.0%). Furthermore, we only evaluated 6 locations using AS-OCT. Therefore, it might potentially be possible that CCD occurred in the other locations and thus, was missed in our current investigation. Based on these findings, the possibility cannot be ruled out that we may have underestimated the manifestation frequency of CCD after μLOT. Third, in our present study, the follow-up period in our patients was short. It has been previously reported that within 3 months after surgery all patients were found to no longer have any CCD, with the postoperative CCD also not affecting the later IOP values until 12 months (12). In addition, it has been shown that if the CCD does not disappear, this is related to the occurrence of persistent hypotony (20). In our current study, we found that within 1 month, the CCD had disappeared in all cases. Based on these overall findings, it is possible that the early postoperative period CCD does not have any effect on the postoperative results after long-term follow-up periods. Furthermore, this was retrospective study and only evaluated a relatively small number of cases. Moreover, some clinical data was missing. For example, in many patients, the gonioscopic examinations were not always done within 2 months. In these cases, gonioscopic findings were only evaluated when the IOP was elevated. Additional multi-center and prospective studies will need to be conducted in order to support the present results.

## Conclusion

In conclusion, after performing μLOT in 62 eyes, CCD occurred in 18 eyes (29.0%) with a subsequent disappearance within 1 month postoperatively in all eyes. With regard to the postoperative IOP, our results showed that there was no significant difference between the CCD group and non-CCD group. However, after the surgery there was a possible transient effect of the lowering of the IOP when there was detection of a connection between the anterior chamber and CCD. After undergoing the μLOT procedure, the presence of a thinner CCT appears to be a risk factor for the development of CCD.

## Data availability statement

The raw data supporting the conclusions of this article will be made available by the authors, without undue reservation.

## Ethics statement

The studies involving human participants were reviewed and approved by Institutional Review Board of the Hiroshima University. The patients/participants provided their written informed consent to participate in this study.

## Author contributions

KH contributed to conception and design of the study. FM, HOn, and KH organized the database. FM and KH performed statistical analysis. FM wrote the first draft of the manuscript. KH and YK wrote sections of the manuscript. All authors contributed to manuscript revision, read, and approved the submitted version.

## References

[1] Ellingsen BA Grant WM. Influence of intraocular pressure and trabeculotomy on aqueous outflow in enucleated monkey eyes. *Invest Ophthalmol Vis Sci.* (1971) 10:705–9. 4999351

[2] Ellingsen BA Grant WM. Trabeculotomy and sinusotomy in enucleated human eyes. *Invest Ophthalmol Vis Sci.* (1972) 11:21–8. 5006959

[3] Tanihara H Honjo M Inatani M Honda Y Ogino N Ueno S Trabeculotomy combined with phacoemulsification and implantation of an intraocular lens for the treatment of primary open-angle glaucoma and coexisting cataract. *Ophthalmic Surg Laesrs.* (1997) 28:810–7. 9336773

[4] Francis BA Singh K Lin SC Hodapp E Jampel HD Samples JR Novel glaucoma procedures: a report by the American academy of ophthalmology. *Ophthalmology.* (2011) 118:1466–80. 10.1016/j.ophtha.2011.03.028 21724045

[5] Caprioli J Kim JH Friedman DS Kiang T Moster MR Parrish RK II Special commentary: supporting innovation for safe and effective minimally invasive glaucoma surgery: summary of a joint meeting of the American glaucoma society and the food and drug administration. Washington, DC, February 26, 2014. *Ophthalmology.* (2015) 122:1795–801. 10.1016/j.ophtha.2015.02.029 25881513

[6] Seibold LK Soohoo JR Ammar DA Kahook MY. Preclinical investigation of ab interno trabeculectomy using a novel dual-blade device. *Am J Ophthalmol.* (2013) 155:524–9. 10.1016/j.ajo.2012.09.023 23218696

[7] Sato T Kawaji T Hirata A Mizoguchi T. 360-degree suture trabeculotomy ab interno to treat open-angle glaucoma: 2-year outcomes. *Clin Ophthalmol.* (2018) 12:915–23. 10.1136/bmjophth-2018-000159 29844656 PMC5963827

[8] Tanito M Sano I Ikeda Y Fujihara E. Shore-term results of microhook ab interno trabeculotomy, a novel minimally invasive glaucoma surgery in Japanese eyes: initial case series. *Acta Ophthalmol.* (2017) 95:e354–60. 10.1111/aos.13288 27805318

[9] Sit AJ McLaren JW. Measurement of episcleral venous pressure. *Exp Eye Res.* (2011) 93:291–8. 10.1016/j.exer.2011.05.003 21621536

[10] Filippopoulos T Rhee DJ. Novel surgical procedure in glaucoma: advances in penetrating glaucoma surgery. *Curr Opin Ophthalmol.* (2008) 19:149–54. 10.1097/ICU.0b013e3282f4f49e 18301289

[11] Akagi T Nakano E Nakanishi H Uji A Yoshimura N. Transient ciliochoroidal detachment after ab interno trabeculotomy for open-angle glaucoma: a prospective anterior-segment optical coherence tomography study. *JAMA Ophthalmol.* (2016) 134:304–11. 10.1001/jamaophthalmol.2015.5765 26823200

[12] Sato T Kawaji T Hirata A. Transient ciliocoroidal detachment after 360-degree suture trabeculotomy ab interno for open-angle glaucoma: 12-month follow-up. *Eye.* (2019) 33:1081–9. 10.1038/s41433-019-0375-5 30792522 PMC6707293

[13] Aoki R Hirooka K Goda E Yuasa Y Okumichi H Onoe H Comparison of surgical outcomes between microhook ab interno trabeculotomy and goniotomy with the Kahook Dual Blade in combination with phacoemulsification: a retrospective, comparative case series. *Adv Ther.* (2021) 38:329–36. 10.1007/s12325-020-01543-3 33113099

[14] Okada N Hirooka K Onoe H Murakami Y Okumichi H Kiuchi Y. Comparison of efficacy between 120° and 180° Schlemm’s canal incision microhook ab interno trabeculotomy. *J Clin Med.* (2021) 10:3181. 10.3390/jcm10143181 34300347 PMC8304718

[15] Schrieber C Liu Y. Choroidal effusions after glaucoma surgery. *Curr Opin Ophthalmol.* (2015) 26:134–42. 10.1097/ICU.0000000000000131 25643198

[16] Sabti K Lindley SK Mansour M Discepola M. Uveal effusion after cataract surgery: an echographic study. *Ophthalmology.* (2001) 108:100–3. 10.1016/s0161-6420(00)00414-0 11150272

[17] Benson WE. Posterior scleritis. *Surv Ophthalmol.* (1988) 32:297–316. 10.1016/0039-6257(88)90093-8 3043740

[18] Damico FM Kiss S Young LH. Vogt-koyanagi-harada diseases. *Semin Ophthalmol.* (2005) 20:183–90. 10.1080/08820530500232126 16282153

[19] Pujari A Selvan H Behera AK Gagrani M Kapoor S Dada T. The probable mechanism of traumatic angle recession and cyclodialysis. *J Glaucoma.* (2020) 29:67–70. 10.1097/IJG.0000000000001358 31460884

[20] Ishida A Mochiji M Manabe K Matsuoka Y Tanito M. Persistent hypotony and annular ciliochoroidal detachment after microhook ab interno trabeculotomy. *J Glaucoma.* (2020) 29:807–12. 10.1097/IJG.0000000000001560 32496462

[21] Pensyl D Sullivan-Mee M Torres-Monte M Halverson K Qualls C. Combining corneal hysteresis with central corneal thickness and intraocular pressure for glaucoma risk assessment. *Eye.* (2012) 26:1349–56. 10.1038/eye.2012.164 22878449 PMC3470051

[22] Aktas Z Ucgul AY Segawa A. Transient myopia secondary to supraciliary effusion: unusual complication after an uneventful prolene gonioscopy-assisted transluminal trabeculotomy. *J Glaucoma.* (2020) 29:e60–3. 10.1097/IJG.0000000000001531 32398587

[23] Paz T Rappoport D Hilely A Leiba H. Bilateral transient myopia with sulfasalazine treatment. *Clin Med Insights Case Rep.* (2019) 12:1179547619855388. 10.1177/1179547619855388 31258341 PMC6585251

